# The PML1-WDR5 axis regulates H3K4me3 marks and promotes stemness of estrogen receptor-positive breast cancer

**DOI:** 10.1038/s41418-024-01294-6

**Published:** 2024-04-16

**Authors:** Chun-Peng Pai, Han Wang, Darcie D. Seachrist, Neel Agarwal, Joshua A. Adams, Zhenghao Liu, Ruth A. Keri, Kaixiang Cao, William P. Schiemann, Hung-Ying Kao

**Affiliations:** 1 Departments of Biochemistry, Case Western Reserve University School of Medicine, Cleveland, OH 44106 USA; 2 Department of Cancer Biology, Cleveland Clinic Lerner Research Institute, Cleveland, OH 44195 USA; 3 Case Comprehensive Cancer Center, Case Western Reserve University, Cleveland, OH 44106 USA; 4 Departments of Molecular Medicine, Case Western Reserve University School of Medicine, Cleveland, OH 44106 USA

**Keywords:** Epigenetics, Oncogenes

## Abstract

The alternative splicing of *PML* precursor mRNA gives rise to various *PML* isoforms, yet their expression profile in breast cancer cells remains uncharted. We discovered that PML1 is the most abundant isoform in all breast cancer subtypes, and its expression is associated with unfavorable prognosis in estrogen receptor-positive (ER+) breast cancers. *PML* depletion reduces cell proliferation, invasion, and stemness, while heterologous PML1 expression augments these processes and fuels tumor growth and resistance to fulvestrant, an FDA-approved drug for ER+ breast cancer, in a mouse model. Moreover, PML1, rather than the well-known tumor suppressor isoform PML4, rescues the proliferation of *PML* knockdown cells. ChIP-seq analysis reveals significant overlap between PML-, ER-, and Myc-bound promoters, suggesting their coordinated regulation of target gene expression, including genes involved in breast cancer stem cells (BCSCs), such as *JAG1*, *KLF4*, *YAP1*, *SNAI1*, and *MYC*. Loss of *PML* reduces BCSC-related gene expression, and exogenous PML1 expression elevates their expression. Consistently, PML1 restores the association of PML with these promoters in *PML*-depleted cells. We identified a novel association between PML1 and WDR5, a key component of H3K4 methyltransferase (HMTs) complexes that catalyze H3K4me1 and H3K4me3. ChIP-seq analyses showed that the loss of *PML1* reduces H3K4me3 in numerous loci, including BCSC-associated gene promoters. Additionally, PML1, not PML4, re-establishes the H3K4me3 mark on these promoters in *PML*-depleted cells. Significantly, PML1 is essential for recruiting WDR5, MLL1, and MLL2 to these gene promoters. Inactivating WDR5 by knockdown or inhibitors phenocopies the effects of PML1 loss, reducing BCSC-related gene expression and tumorsphere formation and enhancing fulvestrant’s anticancer activity. Our findings challenge the conventional understanding of PML as a tumor suppressor, redefine its role as a promoter of tumor growth in breast cancer, and offer new insights into the unique roles of PML isoforms in breast cancer.

## Introduction

The PML protein has diverse cellular functions, including regulating cell-cycle progression, DNA damage responses, and transcription; it also plays important roles in governing immunity, metabolism, and tumorigenesis [1–7]. PML protein is primarily localized in the nucleoplasm and DNA-free sub-nuclear compartments known as PML nuclear bodies (NBs) [8–10], which may indirectly regulate transcription by sequestering transcription factors or serving as a platform protein for transcription factor modification [11, 12]. Several studies have suggested that PML binds to chromatin, indicating a direct role in transcriptional regulation [6, 13, 14]. However, a systematic analysis of the global PML-bound promoters is lacking.

The notion that *PML* is a tumor suppressor gene was mainly based on studies of the *PML4* isoform [12, 15–17] and earlier clinical investigations [17]. Recent studies have revealed a more complex role for PML in cancer. Knockdown of *PML* inhibits the proliferation of estrogen receptor-positive (ER+) breast cancer [18] and ovarian cancer cells [19] and reduces tumor growth in mouse xenograft models of triple-negative breast cancer (TNBC) [20–22] and glioblastoma [23]. Interestingly, the PML-reducing agent arsenic trioxide (ATO), an FDA-approved drug for treating acute promyelocytic leukemia, is an effective agent in inhibiting tumor growth of glioblastoma [23, 24] and TNBCs [22]. These paradoxical findings underscore the need to revisit our understanding of PML’s role in tumorigenesis.

The *PML* precursor mRNA undergoes alternative splicing, resulting in multiple isoforms, and the expression patterns of different *PML* isoforms in cancerous tissues and their specific contribution to tumorigenesis remain unknown. This study examined the expression profiles of *PML* isoforms in normal and malignant breast cells and tissues. We found that *PML1* is the most abundant isoform expressed in ER+ breast tumors and cancer cell lines, with the increased *PML1* mRNA associated with poor prognosis of luminal breast cancer patients. Significantly, a recent clinical study revealed that the *PML* gene is amplified in 14% of ER+ metastatic breast cancer (MBC) [25]. We also showed that the loss of *PML* inhibits the stemness of ER+ breast cancer cells, with elevated PML1 expression driving breast cancer stemness, tumor growth, and therapy resistance in xenograft mouse models.

To further understand the mechanism by which PML1 promotes breast tumorigenesis and stemness, we analyzed ChIP-seq data. We found that PML, Myc, and ER bind many common gene promoters, including those encoding breast cancer stem cell (BCSC)-related genes, such as *JAG1* [26], *KLF4* [27], *YAP1* [28], *SNAI1* [29], and *MYC* [30]. PML1 promotes the expression of both Myc and ER target genes, thereby increasing ER+ breast cancer cell stemness. We also discovered that PML1 associates with WDR5 and regulates H3K4 tri-methylation (H3K4me3), and the inactivation of WDR5 reduces breast cancer cell stemness and related gene expression and enhances the anticancer activity of fulvestrant. Mechanistically, PML is essential for recruiting WDR5, MLL1, and MLL2 to the stemness gene promoters, thus regulating the H3K4me3 marks at these loci. Our findings redefine the role of PML, shifting its characterization from a tumor suppressor to a promoter, and highlight the pivotal function of the PML1-WDR5 axis in regulating breast cancer cell stemness and drug resistance.

## Results

### PML1 is the most abundant isoform in estrogen receptor-positive (ER+) breast tumors

**Fig. 1:**
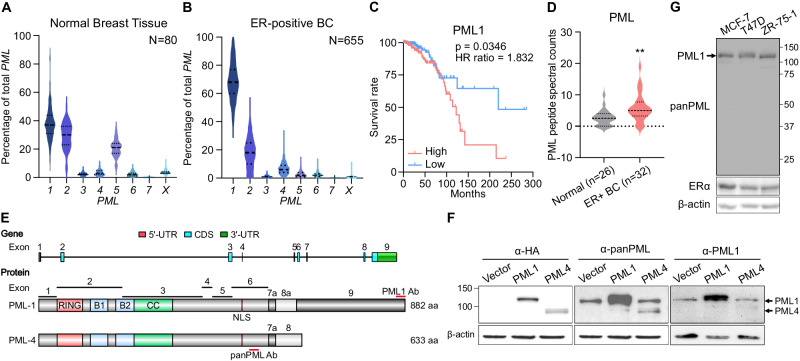
PML1 is the predominant isoform in HR+BC cells and patients. Relative abundance of eight *PML* protein-coding isoforms (UCSC genome browser, hg38) in normal breast (**A**) and ER+ breast cancer tissues (**B**). The nomenclatures of each isoform, defined by a unique exon and may include more than one sub-isoform, are previously described [18]. **C** A correlation of *PML1* mRNA levels with survival rates in luminal BC. Patients whose *PML1* mRNA ratio is at the top 30% (high, *N* = 196) or bottom 30% of all patients were evaluated for outcomes. **D** PML protein abundance is elevated in ER+ breast tumors. **E** The structure of *PML* gene (top) and exon-encoded domains (bottom) in PML1 and PML4. Antigens for panPML and PML1-specific antibodies are indicated. PML1 is the largest isoform. **F** MCF-7 cell lysates expressing HA-PML1 or HA-PML4 probed with HA (lanes 1–3), pan-PML (lanes 4–6), or PML1-specific antibodies (lanes 7–9). **G** PML1 is the predominant isoform in ER+ breast cancer cells. A panPML antibody was used to detect all PML protein isoforms.

To better understand the expression patterns of *PML* isoforms in breast cancer patients, we interrogated RNA-seq datasets from normal breast tissues (GTEx) and breast tumors (TCGA). Our results demonstrate that the total *PML* transcript expression is significantly elevated across all breast cancer subtypes compared to normal tissues (Fig. S1). *PML1* mRNA is the predominant isoform in normal breast tissues and ER+ breast tumors. Moreover, *PML1* abundance shifts dramatically from ~38% in normal tissues (Fig. 1A) to ~67% in ER+ tumors (Fig. 1B), while that of *PML2* mRNA is expressed at a lower level than *PML1* in both normal (~30%) and malignant breast tissues (~20%). *PML4*, which encodes an extensively studied tumor suppressor, is expressed at a much lower level (~7%). Moreover, higher *PML1* mRNA levels are associated with poor prognosis of ER+ breast cancer patients (Fig. 1C), but there was no correlation between the expression of other *PML* isoforms and prognosis (Fig. S2A). Furthermore, the total PML protein abundance is elevated in ER+ breast tumors (Fig. 1D). We also observed a trend in which higher PML protein abundance correlates with poor prognosis (Fig. S2B). PML1 and PML4 proteins share the first 620 amino acids, with PML4 containing a 13 a.a. unique C-terminus and PML1 possessing an additional 262 a.a (Fig. 1E). To better understand the role of PML1 in breast cancer, we generated a PML1-specific antibody. We confirmed that PML1 and PML4 proteins migrate around 130 kDa and 100 kDa, respectively (Fig. 1F) and that PML1 is the predominant isoform in ER+/HER2− breast cancer cell lines, including MCF-7, T47D, and ZR-75-1 cells (Fig. 1G). These findings suggest that PML1 is the most abundant isoform in breast cancer, and its high expression may be a potential biomarker for poor prognosis for ER+ breast cancer.

### PML1 promotes cancer phenotypes and fulvestrant resistance

**Fig. 2:**
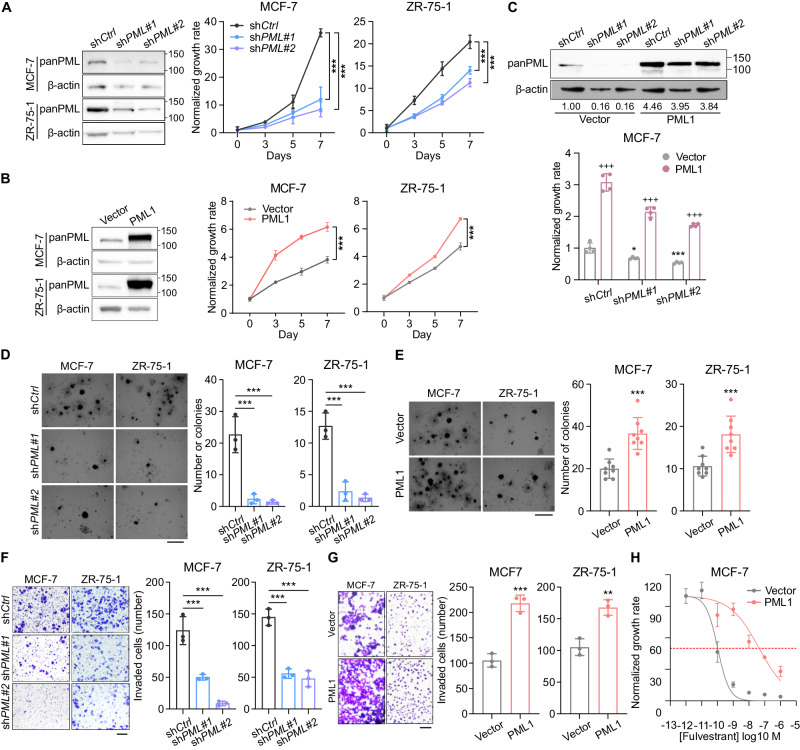
The role of PML in proliferation, migration, and invasion of ER+ breast cancer cells. The effects of transient *PML* knockdown (**A**, **D**, **F**) and PML1 overexpression (**B**, **E**, **G**) on proliferation (**A**, **B**), colony formation (**D**, **E**), and invasion (**F**, **G**) of MCF-7 and ZR75-1 cells. The effects of PML1 overexpression on proliferation (**B**), colony formation (**E**), and invasion (**G**) of MCF-7 and ZR75-1 cells. **C** Exogenous PML1 rescues the proliferation defect of *PML* knockdown cells. **H** PML1 increases fulvestrant IC_50_. The data were analyzed using one-way ANOVA. PML1-OE experiments were performed with *N* = 8, while the rest had *N* = 3.

Our previous study demonstrated that the ectopic overexpression of PML4 inhibits the proliferation, migration, and invasion of MCF-7 cells [18]. We expand our studies by investigating the effects of PML on another ER+ breast cancer cell line, ZR-75-1. Our results showed that the knockdown of *PML* reduces the proliferation (Fig. 2A), colony formation (Fig. 2D), and invasion (Fig. 2F) of MCF-7 and ZR-75-1 cells, while *PML1* overexpression has the opposite effect (Fig. 2B, E, and G). Furthermore, MCF-7-HA-PML1 cells, which express virally transduced HA-PML1, exhibit a significant increase in the IC_50_ (4.499e-008M) for fulvestrant, compared to control cells (1.046e-010M) (Fig. 2H), indicating that higher PML1 expression promotes fulvestrant resistance. This result is consistent with a recent clinical study indicating that the *PML* gene is amplified in 14% of ER + MBC [25] (Fig. S3). Moreover, exogenous PML1 rescues the proliferation of *PML* knockdown cells (Fig. 2C), but PML4 does not (Fig. S4A). Additionally, PML2 inhibits the proliferation and breast cancer cell stemness (Fig. S4B), indicating that PML2 and PML1 have the opposite effects on breast cancer cells. These results suggest that PML isoforms play distinct roles in breast cancer development and progression and that PML1 may play a role in fulvestrant resistance.

### PML1 binds and positively regulates stemness gene promoters and promotes breast cancer stem-like cell (BCSC) populations

**Fig. 3:**
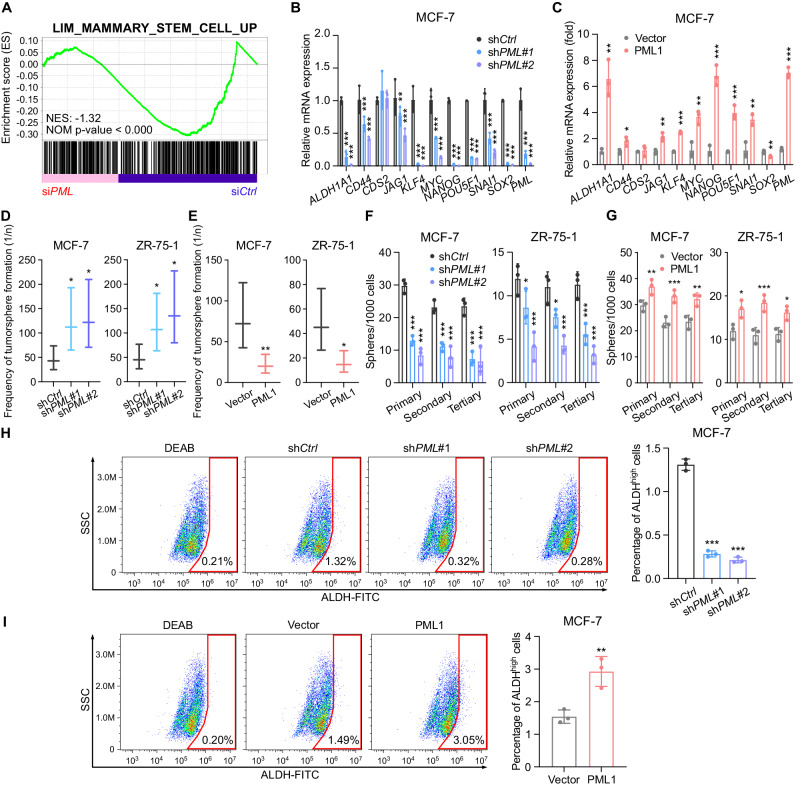
PML1 promotes cancer cell stemness in ER+ breast cancer Cells. **A** PML regulates a subset of CSC-related gene promoters, which are enriched in the Mammary_Stem_Cell_Up signature. *PML* knockdown in ER+ breast cancer cells leads to a significant decrease in the expression of several CSC-related genes (**B**), while PML1 overexpression enhances stemness-related gene expression in MCF-7 cells (**C**). *PML* knockdown reduces the population of CSCs (**D**), while overexpression of exogenous PML1 increases the CSC population in ER+ breast cancer cells (**E**). *PML* knockdown reduces tertiary mammosphere formation (**F**), a characteristic of CSCs, whereas PML1 overexpression enhances tertiary mammosphere formation (**G**). **F** PML knockdown reduces the population of ALDH^high^ cells, indicating decreased stem cell-like features (**H**), while PML1 overexpression increases the ALDH^high^ cell population in ER+ breast cancer cells (**I**). The data (**B**–**G**) were analyzed by two-way ANOVA, and data (**H**–**I**) were analyzed by one-way ANOVA, with all experiments conducted with three biological replicates.

The observations that PML1 promotes fulvestrant resistance and invasion of breast cancer cells prompted us to investigate PML1’s role in cancer cell stemness. Gene Set Enrichment Analysis (GSEA) revealed that affected genes in *PML* knockdown microarray gene expression study are enriched for genes upregulated in the Mammary_Stem_Cell_Up signature [31] (Fig. 3A), suggesting PML’s role in CSC regulation. Analyses of PML ChIP-seq data in MCF-7 cells revealed that PML binds to more than half of the BCSC-associated gene promoters (Table S3). Knockdown of *PML1* significantly reduced the expression of a subset of BCSC-related genes (Fig. 3B), while overexpression of PML1 increased their expression (Fig. 3C). Moreover, *PML1* knockdown reduced the frequency of BCSCs in extreme limiting dilution assays (ELDAs) (Fig. 3D) and tertiary tumorsphere-formation assays (Figs. 3F, S5), while PML1 overexpression had the opposite effect (Figs. 3E, G, and S5). FACS analyses further showed that *PML* knockdown reduced the ALDH^high^ cell population, while overexpression of PML1 increased it (Fig. 3H, I). These results suggest that PML1 promotes the stemness of breast cancer cells.

### PML1 promotes tumor growth and fulvestrant resistance in a xenograft animal model

**Fig. 4:**
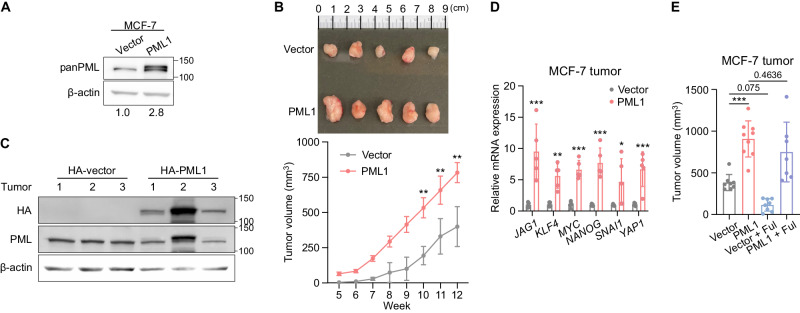
PML1 promotes tumor growth and fulvestrant resistance of MCF-7 cells. **A** HA-PML1 stably expressing MCF7 cells (left) generated larger tumors (right) following injection of the same number of viable tumor cells. Images representing tumor size from MCF-7 cells expressing PML1 and vector. **B** Quantification of tumor growth in mice injected with PML1-overexpressing or empty vector control cells was performed with a sample size of *N* = 10 in each group. **C** The western blot showing PML1 overexpression in tumors harvested from mice after 12 weeks of growth. **D** The effect of PML1 on stemness gene expression in tumors was assessed with a sample size of *N* = 9 in each group. **E** Tumor size was quantified at weeks 0 and 8 in mice bearing PML1 or empty vector tumors after fulvestrant treatment, with a sample size of *N* = 7–9 for each group. The data were analyzed using Two-way ANOVA.

Next, we determined the effects of PML1 on the tumor growth of MCF-7 cells. Our results showed that animals xenografted with MCF-7-HA-PML1 cells developed significantly larger tumors than those with control cells (Fig. 4A, B). These findings suggest that PML1 plays a crucial role in promoting tumor growth in breast cancer. HA-PML1 protein expression is confirmed by western blots in tumors stably express HA-PML1 (Fig. 4C), and HA-PML1-expressing tumors show elevated BCSC-related gene expression (Fig. 4D) Lastly, tumors generated with cells expressing MCF-7-HA-PML1 were resistant to fulvestrant (Fig. 4E). These observations are consistent with the fact that 14% of ER + MBC have the *PML* gene amplification. These findings suggest that PML1 is crucial in promoting tumor growth and fulvestrant resistance in breast cancer.

### ChIP-seq analyses reveal crosstalk between PML1, ER, and Myc-bound promoters

**Fig. 5:**
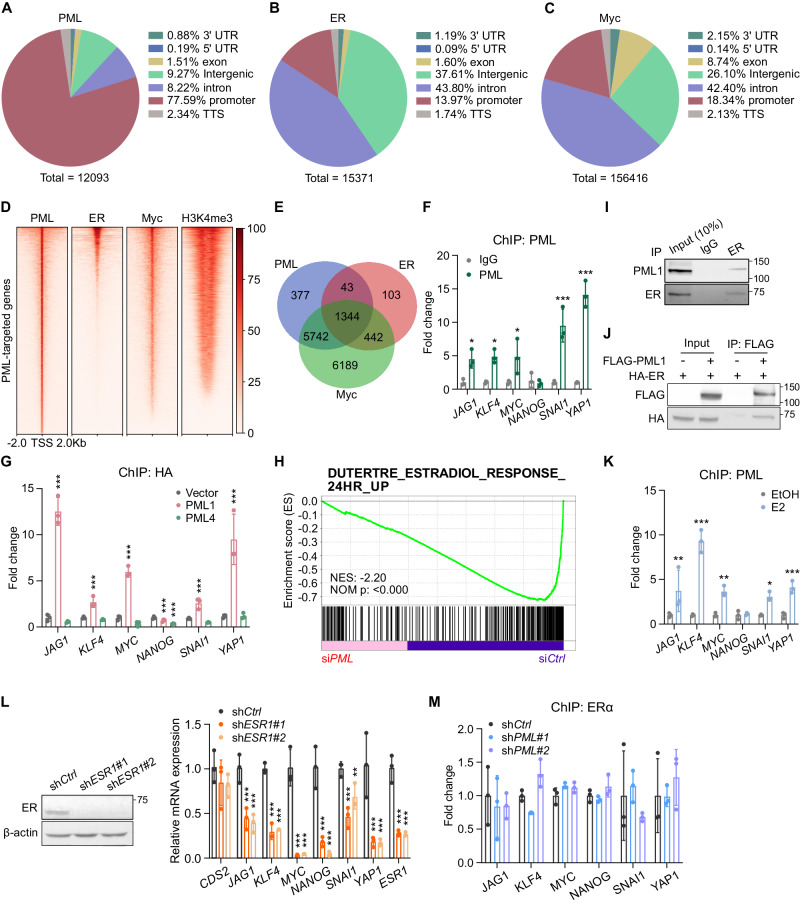
PML modulates stemness-related gene regulation and ER signaling in breast cancer. Genomic distribution of PML- (**A**), ER- (**B**), and Myc-binding sites (**C**) ON stemness-related gene promoters. The promoter region is defined as −1 kb to +1 kb from the transcription start site (TSS), and the transcription terminator (TTS) is defined as −100 bp to +1 kb from the termination site. **D** Heatmaps displaying the binding sites of PML, ER, and Myc on PML-bound promoters. **E** Venn diagram showing the numbers of overlapped PML, ER, and Myc-bound promoters. **F** ChIP-qPCR results demonstrating PML binds several BCSC-related gene promoters. **G** PML1, not PML4, binds promoters of BCSC-related genes. PML knockdown cells were subsequently transfected with vector alone, PML1, or PML4 expression plasmids. **H** Enrichment plot demonstrates PML knockdown target genes enriched in DUTERTRE_ESTRADIOL_RESPONSE_24HR_UP signature. Endogenous (**I**) and transfected PML1 (**J**) interact with ER in MCF-7 cells. **K** Estradiol induces PML associations with BCSC-related gene promoters. **L** ESR1 knockdown reduces BCSC-related gene expression. **M** PML loss does not impact ER-chromatin associations. Data were analyzed by Two-way ANOVA. Each experiment with *N* = 3 in each group.

Previous reports have shown that the Myc transcription factor regulates the expression of a subset of stemness genes [32] and Myc interacts with PML4 [33]. Analyses of ChIP-seq data for PML, Myc, and ER revealed that most PML-binding sites (~77%) are in promoter regions, which account for 23% of protein-coding gene promoters (Fig. 5A and E). In contrast, less than 14% of the ER-binding sites are in promoter regions, while ~80% are in intergenic regions or introns (Fig. 5B). Interestingly, most Myc-binding sites are in intergenic regions or introns (Fig. 5C). Focusing on PML-bound promoters (Fig. 5D), we found that PML and ER bind 1387 common promoters (Fig. 5D, E), which accounts for ~70% of ER- and ~18% of PML1-bound promoters, respectively (Figs. 5E, S6A). The top-ranked consensus sequence among PML1 and ER commonly bound promoters is an estrogen-response element (ERE) half-site, -AGGTCA- (Fig. S6B). Myc binds ~94% of PML-bound promoters in MCF-7 cells (Fig. 5E). In fact, microarray analyses [34] suggest that affected genes in *PML* knockdown cells are enriched in Myc-targeted genes (Fig. S6C). Furthermore, ChIP-seq analyses suggest that PML1, Myc, and ER bind several BCSC-related gene promoters (Table S3), including *JAG1*, *KLF4*, *MYC*, *SNAI1*, and *YAP1* (Fig. S7). Using ChIP-qPCR, we confirmed that PML binds these promoters but not *NANOG* (Fig. 5F) and that PML1, not PML4, binds these promoters (Fig. 5G). These analyses suggest that PML, Myc, and ER regulate gene expression in BCSCs by binding to common promoters. Moreover, microarray gene expression analyses [34] indicate that PML target genes are enriched in estradiol-responsive genes [35] (Fig. 5H). Proximity ligation assays (PLA) showed that endogenous PML and ER interact (Fig. S8). Furthermore, Coimmunoprecipitation demonstrated endogenous and exogenous PML1 and ER interact (Fig. 5I, J), and the recruitment of PML1 to BCSC-related gene promoters is induced upon E2 treatment (Fig. 5K), indicating a potential role of PML1 in E2-induced ER-target gene expression. Furthermore, the knockdown of *ESR1* significantly reduces the expression of stemness-related genes, phenocopying the effects of *PML1* knockdown (Fig. 5L). However, the loss of *PML1* had little or no effect on the ER binding to these promoters (Fig. 5M), suggesting PML1 regulates ER target gene expression without affecting ER binding to the promoters.

### PML1 interacts with WDR5, a core subunit of the histone H3 lysine 4 methyltransferase (H3K4 HMTs) complexes

**Fig. 6:**
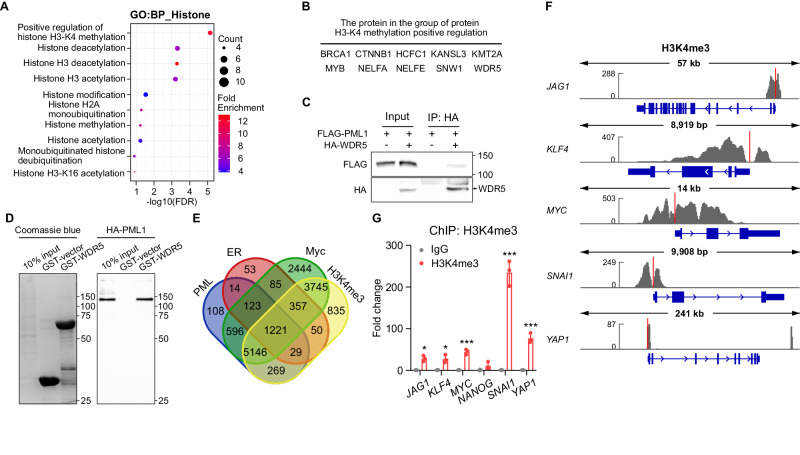
PML crosstalk with WDR5 to promote H3K4me3 at BCSC-related gene promoters. **A** In silico analyses of H3K4 methylation-related, PML-interacting proteins. **B** A list of putative PML-interacting proteins that promote H3K4 methylation (top). **C** PML1 and WDR5 interact in MCF-7 cells, as shown by coimmunoprecipitation assays (bottom). **D** GST-WDR5 binds HA-PML1 expressed in MCF-7 cells. Left: Coomassie staining, right: anti-HA Western blotting. **E** A Venn diagram shows the overlapped promoters bound by PML, ER, Myc, and the H3K4me3 mark. **F** ChIP-seq tracks show the distribution of the H3K4me3 mark on *JAG1*, *KLF4*, *MYC*, *SNAI1*, and *YAP1* genes. **G** The stemness gene promoters are marked with H3K4me3.

To investigate the underlying mechanism of how PML1 promotes ER and Myc transcriptional activity, we utilized an *in-silico* approach to screen for PML1-interacting proteins, which identified several putative PML-interacting proteins involved in histone modification (Fig. 6A), including proteins involved in histone H3K4 methylation (Fig. 6B), such as WDR5 [36]. WDR5 is a core subunit of all four MLL1-4 histone methyltransferase complexes. These complexes catalyze the methylation of histone H3 lysine 4 (H3K4), with MLL1/2-containing complexes responsible for H3K4me3 and MLL3/4-containing complexes catalyzing H3K4me1 [37]. Interestingly, in silico analyses also suggest an association of PML with MLL1. Using co-immunoprecipitation (Fig. 6C), GST pulldown assays (Fig. 6D), and PLA (Fig. S8), we showed that PML1 and WDR5 physically interact. Furthermore, we analyzed the ChIP-seq database to examine the H3K4me3 status of PML1-, ER-, and Myc-bound promoters. Our analysis revealed that H3K4me3 marks ~88% of PML-bound promoters, and ~90% of PML, Myc, and ER commonly-bound promoters are enriched in H3K4me3 (Fig. 6E). Specifically, several BCSC-related genes described above are enriched with the H3K4me3 mark (Fig. 6F, G).

**Fig. 7:**
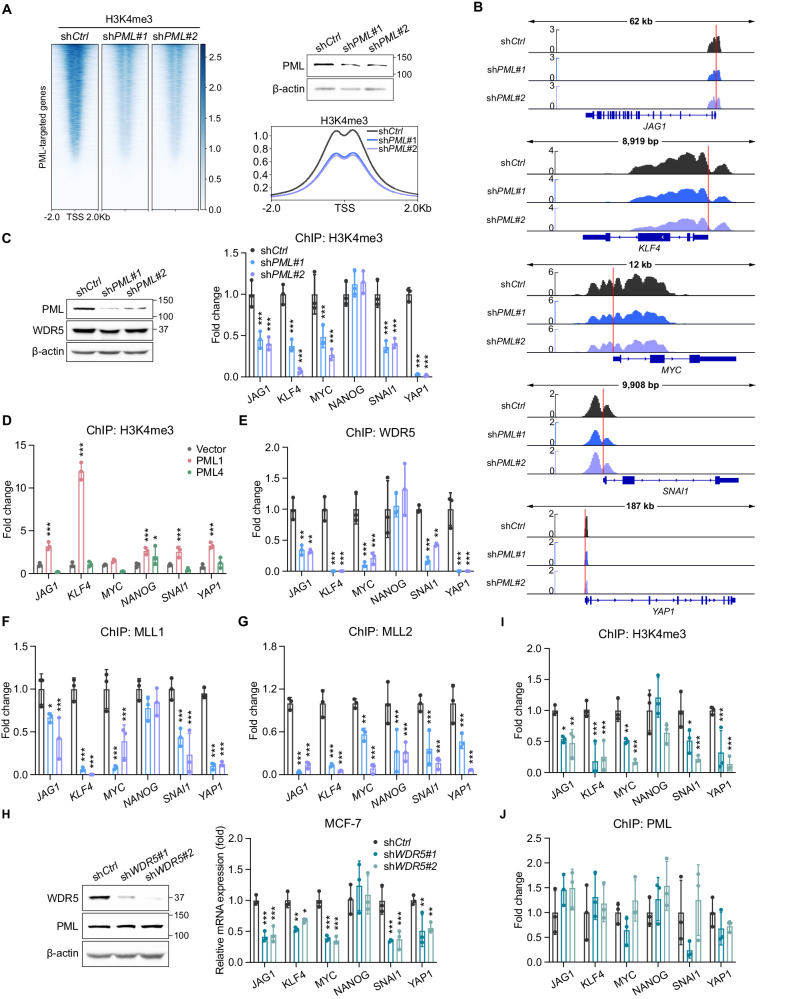
PML1 regulates H3K4me3 marks and is critical in recruiting WDR5, MLL1, and MLL2. **A** Heatmap illustrating changes in H3K4me3 binding sites across PML-bound promoters following PML loss. Heatmaps show representative data from triplicate experiments, while meta-plots illustrate outcomes from triplicate experiments. **B** ChIP-seq tracks reveal H3K4me3 distribution on *JAG1*, *KLF4*, *MYC*, *SNAI1*, and *YAP1* genes, with and without PML-knockdown, while genomic tracks depict aggregated outcomes from triplicate experiments. **C** Loss of *PML* significantly reduces the H3K4me3 mark. **D** PML1, not PML4, restores H3K4me3 marks on BCSC-related gene promoters in *PML* knockdown cells. PML1 is required for the associations of WDR5 (**E**), MLL1 (**F**), and MLL2 (**G**) with the stemness gene promoters. Loss of *WDR5* reduces the expression of the stemness genes (**H**) and the H3K4me3 (**I**) mark on their promoters but has little or no effect on PML associations with these gene promoters (**J**). The data were analyzed using Two-way ANOVA. Each experiment was performed with *N* = 3 in each group.

To interrogate the role of PML1 in regulating global H3K4me3 across gene promoters, we performed *PML* knockdown followed by ChIP-seq, which showed that PML1 regulates H3K4me3 levels at numerous gene promoters (Fig. 7A), including gene loci associated with BCSCs (Fig. 7B). Additionally, the H3K4me3 patterns we observed on these promoters align well with publicly accessible data (Fig. S7). Importantly, ChIP-qPCR confirmed that the loss of *PML1* significantly reduced the H3K4me3 mark on BCSC-related gene promoters (Fig. 7C). Because PML1 and PML4 contain the WDR5-interacting domain, we examined whether PML1 and PML4 can restore the H3K4me3 mark in *PML* knockdown cells, and our data demonstrated that PML1, not PML4, re-establishes the H3K4me3 mark in *PML* knockdown cells (Fig. 7D). Furthermore, the loss of *PML1* significantly reduced the associations of WDR5 (Fig. 7E), MLL1 (Fig. 7F), and MLL2 (Fig. 7G) with stemness gene promoters. We further investigated whether WDR5 is required for PML associations with these promoters and found that knockdown of *WDR5* markedly reduces the expression of the BCSC-related genes (Fig. 7H) and the H3K4me3 mark (Fig. 7I) but has little or no effect on PML1 associations with these promoters (Fig. 7J). These data suggest that PML1 promotes ER and Myc transcriptional activity through its interaction with WDR5 and the subsequent enrichment of the H3K4me3 mark at target gene promoters.

### Inactivation of WDR5 enhances the effectiveness of fulvestrant in PML1-overexpressing cells

**Fig. 8:**
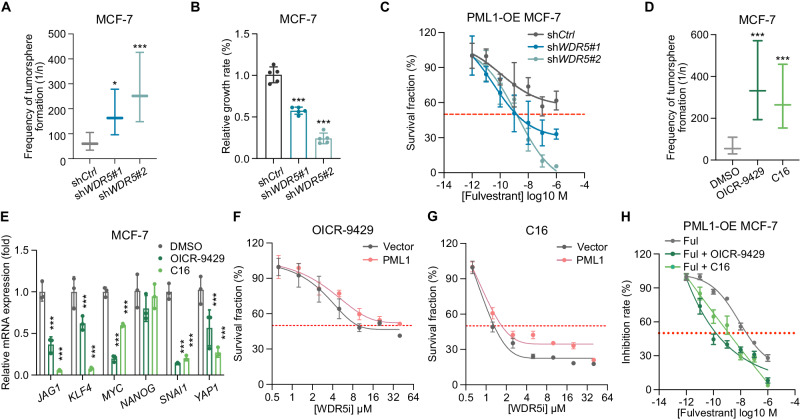
Inactivation of WDR5 by knockdown or WDR5i inhibits stemness and proliferation of MCF-7 cells and promotes fulvestrant’s anticancer activity. Knockdown of WDR5 reduces stem cell populations (**A**) and the proliferation (**B**), and fulvestrant’s IC_50_ (**C**) in MCF-7 cells. OICR-9429 and C16 treatments reduce stem cell population (**D**) and stemness-related gene expression (**E**) of MCF-7 cells. PML1 rescues the proliferation of OICR-9429- (**F**) and C16-treated cells (**G**). OICR-9429 and C16 significantly enhance fulvestrant’s anticancer activity of PML1 stably expressing MCF-7 cells (**H**). IC_50_ for Ful, Ful plus OICR9429, and Ful plus C16 are 2.511e-008, 1.120e-010, and 1.447e-009, respectively. The IC_20_ of OICR9429 (2.5 μM) or C16 (1.25 μM) was used in combination with fulvestrant. Most experiments were analyzed using Two-way ANOVA, whereas IC_50_ data were analyzed using Prism 9 for dose-response curve fitting and calculation of IC_50_ values. Each experiment was performed with *N* = 3 in each group.

The data presented above suggests that WDR5 and PML may act together to modulate the expression of stem cell-associated genes and stemness in breast cancer cells. Our results demonstrate that the knockdown of WDR5 leads to a significant decrease in BCSCs population (Figs. 8A and S9) and inhibition of MCF-7 cell proliferation (Fig. 8B). We also found that the knockdown of *WDR5* significantly enhances the anti-proliferation activity of fulvestrant against PML1-overexpressing cells, reducing the IC_50_ from μM to nM (Fig. 8C). We next investigated the effects of pharmacological inhibitors of WDR5, OICR-9429 and compound 16 (C16), on stemness-related gene expression, cell proliferation, and the anticancer activity of fulvestrant. Both inhibitors disrupt the interaction between WDR5 and MLL1 by targeting their interacting sites [38, 39]. Our results demonstrated that both inhibitors effectively reduced the population of BCSCs (Fig. 8D), inhibited the expression of stemness-related genes (Fig. 8E), and suppressed the proliferation of both control and PML1-overexpressing cells (Fig. 8F, G). Furthermore, both inhibitors enhanced the anti-growth activity of fulvestrant (Fig. 8H). These results suggest that the PML1:WDR5 association has functional significance in regulating breast cancer stemness and fulvestrant resistance.

## Discussion

Our study provides compelling evidence that *PML1* promotes the proliferation, migration, and tumor growth of ER+ breast cancer cells. We demonstrated that *PML1* is the most abundant isoform expressed in ER+ breast tumors and plays a critical role in promoting cancer cell stemness and resistance to fulvestrant. In support of our conclusions, a recent clinical study reported that the *PML* gene is amplified in 14% of ER + MBC cases [25], suggesting that elevated PML1 protein promotes metastasis. These observations affirm the notion that rather than functioning as a tumor suppressor, the *PML1* isoform promotes breast tumorigenesis and metastasis. Moreover, we showed that PML1, not PML4, rescues the proliferation and restores H3K4me3 of *PML* knockdown cells. Our findings fill the knowledge gap and help to explain the conflicting data regarding the role of *PML*’s role in tumorigenesis, which we attribute to the limited understanding of the distinct roles and abundance of different *PML* spliced isoforms. Importantly, our study elucidates the underlying molecular mechanism by which PML1 promotes the proliferation and stemness of ER+ breast cancer cells by regulating the stemness gene expression through the recruitment of WDR5 and establishing the H3K4me3 mark.

Despite the lack of statistical significance, we observed the trend of the effect of fulvestrant on MCF-vector tumors (Fig. 4E). The lack of statistical significance could be due to temporal changes in tumor response. We found that tumor growth in the MCF7 vector group was significantly inhibited during the first few weeks of fulvestrant treatment. However, some tumors showed accelerated growth in the weeks before tumor harvest, which may be due to fulvestrant treatment selecting specific cell populations within the tumor that overcome the cytotoxic effects of fulvestrant. Additionally, sustained E2 release throughout fulvestrant treatments may reduce the efficacy of fulvestrant in inhibiting tumor growth.

Contrasting to previous reports that PML4 interacts and inhibits Myc transcription activity [40], our data showed that PML1 positively regulates *MYC* expression and that Myc protein binds to ~94% of PML-bound promoters, underscoring the critical role of Myc in recruiting PML to promoters and promoting cancer cell stemness. A retrospective study of ER+ breast tumors also suggested that the *MYC* gene amplification might contribute to endocrine therapy resistance [41–43]. These observations suggest that PML1 and Myc work together to promote endocrine therapy resistance and highlight the potential of targeting the PML1-Myc axis as a therapeutic strategy for overcoming endocrine therapy resistance in ER+ breast cancer. Future research is needed to elucidate additional mechanisms by which PML1 promotes endocrine therapy resistance, including identifying other potential players.

Our study raises several important questions that warrant further investigation. For example, it is unclear how alternative splicing controls the abundance of different *PML* isoforms and whether this regulation is a general mechanism that operates across different cancer types. Furthermore, our findings suggest that alternative splicing is a critical mechanism regulating tumorigenesis, highlighting the need for further research and a potential strategy to treat breast cancer by targeting aberrant alternative splicing. These findings provide important insights into the complex regulation of *PML* isoforms and their role in breast cancer and lay the groundwork for future investigations into the molecular mechanisms that drive tumorigenesis.

Our study highlights the importance of nucleoplasmic PML, including chromatin-bound PML, in regulating transcription. By interrogating and combining public datasets, we identified over 12,000 PML-binding sites, primarily found in gene promoters. Moreover, PML proteins associate with more than 70% of ER-bound promoters, and loss of PML had little or no effect on ER associations with the promoters, suggesting that PML is recruited to chromatin by sequence-specific transcription factors, such as Myc and ER.

Our data also demonstrate that PML1 promotes transcriptional activation, as evidenced by its requirement for the enrichment of H3K4me3 (~88%) and the recruitment of WDR5, MLL1, and MLL2 on PML-bound promoters. It is worth noting that Myc binds WDR5 [44], and ER interacts with MLL2 [45], implying that WDR5 may have a broader role in regulating transcriptional activation beyond breast tumors. Previous reports suggest that WDR5 expression is a prognostic factor in breast cancer outcomes [46] and a potential therapeutic target [47]. Recent investigations have also linked WDR5 to GBM stemness [48], indicating that it may be a potential target for treating this type of cancer. Overall, our study highlights the importance of WDR5 in PML1-mediated gene expression to promote breast tumor growth and stemness and suggests that targeting the PML1-WDR5 axis may be a promising therapeutic strategy for various cancers.

## Materials and methods

### Cell culture

The HEK293T and MCF-7 cell lines were procured from the American Type Culture Collection (ATCC) and cultured on tissue culture plastic, employing Dulbecco’s Modified Eagle’s medium (DMEM) enriched with 10% fetal bovine serum (FBS) and 50 units/ml Penicillin-Streptomycin Solution (P/S). T47D and ZR-75-1 cells (also from ATCC) were nurtured in RPMI-1640 medium supplemented with 10% FBS and 50 units/ml P/S. In the case of T47D cells, an additional 5 μg/ml insulin was introduced into the medium. Mouse embryonic fibroblast (MEF) cells were generated in-house and cultured in DMEM supplemented with 10% FBS and 50 units/ml P/S. All cell lines were maintained at 37 °C in a 5% CO_2_ incubator. Transient transfections were performed using Lipofectamine 2000 (Thermo Fisher, #11668019) following the manufacturer’s instructions.

### Flow cytometry

MCF-7 and ZR-75-1 cells were dissociated, labeled with antibodies (1–2 μg per 10^6 cells for 1 h), and subsequently resuspended in 1X phosphate-buffered saline (PBS) according to established procedures [49]. The ALDEFLUOR assay was conducted in adherence to the manufacturer’s guidelines, followed by flow cytometry using a BD Accuri C6 Plus Flow Cytometer (BD Biosciences). Electronic gating was configured based on cells stained with the corresponding control (DEAB). Details of the antibodies used are provided in Supplementary Table 1.

### Statistical analysis

The difference in continuous measurements among groups will be determined using a t-test (two groups) assuming unequal variance or ANOVA (more than two groups) followed by Tukey pair-wise comparison procedure. Differences between groups were considered statistically significant at values of *p* ≤ 0.05. Data were depicted as the mean ± SD, using ***p < 0.001 as significance criteria. *p* < 0.05 and *p* < 0.01 are designated by * and **, respectively. The likelihood ratio test and Chi-square test were used to assess the significance.

### Supplementary information

Supplementary Methods and Figure Legends

Supplementary Figures

Supplementary Tables

Original Western Blot

## Data Availability

The datasets generated during and/or analyzed during the current study are available in the GSE255018.
