# Supplementary material for: The PML1-WDR5 axis regulates H3K4me3 marks and promotes stemness of estrogen receptor-positive breast cancer
Source: Cell Death Differ. 2024 Apr 16;31(6):768–78. doi: 10.1038/s41418-024-01294-6 (PMC11164886; doi:10.1038/s41418-024-01294-6)
Supplement: Supplementary file 1 — Supplementary Methods and Figure Legends [file 41418_2024_1294_MOESM1_ESM.pdf]

**Supplementary Information**

**Supplementary Methods**

**Expression plasmids**

The full-length and fragment cDNAs of human PML1 were cloned into the pCMX-1H vector. HA-PML1 was subcloned into the pLVX-IRES-puro (Clontech/Takara Bio, CA, USA). All plasmids were verified by DNA sequencing. Lentivirus was produced in HEK293T cells (ATCC) using the packaging vector pMD2.G (Addgene #12259) and psPAX2 (Addgene #12260). The GST-WDR5 expression plasmid was cloned into pGEX4T-1.

**Lentiviral shRNA transduction**

Sustained knockdown (KD) of PML expression in cells was achieved through the utilization of validated shRNA oligonucleotides incorporated into the lentivector pLKO.1-puro (MISSION shRNA lentiviruses; Sigma-Aldrich, St. Louis, MO) in accordance with the manufacturer's protocol. Specifically, the following shRNAs were employed for knockdown: PML (TRCN00000003867 and TRCN0000355997), WDR5 (TRCN0000431031 and TRCN0000118049), ESR1 (TRCN0000003300 and TRCN0000003301), and a non-target control (SHC005; control shRNA or control KD). Lentiviruses were generated in HEK293T cells by transfection with pMD2.G, psPAX2, and pLKO.1-shRNA vector using Lipofectamine 2000. The viral supernatant was harvested at 48, 72, and 96 hours post-transfection, filtered through a 0.45 μm filter, and stored at -80°C until use. Cells were transduced with a medium-virus soup mixture at a 1:1 ratio, supplemented with 10 μg/mL polybrene, for 24 hours. Following this, the virus-containing medium was replaced with fresh media containing puromycin for a 48-hour selection period. The transduced cells were assessed for knockdown efficiency through RT-PCR and immunoblot analysis prior to subsequent experimentation.

**Tumorsphere Formation assays**

Single-cell suspensions of breast cancer cells were cultivated as spheres in the MammoCult Human Medium Kit (STEMCELL; Catalog #05620). Viable cells were adjusted to the specified cell numbers (ranging from 2000 cells/well to 10 cells/well), with each cell density subjected to six replicates, in 100 μL of medium per well using a

96-well Ultra-Low Attachment Microplate (Corning, AZ, USA, #3474). The cultures were maintained at 37 °C with 5% CO2. Tumorsphere presence was assessed after ten days, and the obtained data were subjected to analysis and visualization using the ELDA software (http://bioinf.Wehi.edu.au/software/elda/index.html) [1]. Each experiment was conducted in triplicate, and results from three independent experiments were comprehensively analyzed.

**Gene Set Enrichment Analysis (GSEA)**

The *PML*-KD mRNA expression profile was analyzed by GSEA using GSEAv4.3.2 software. Signatures M2573, M2156, and M6506 were used to enrich breast LIM_MAMMARY_STEM_CELL_UP(*33*), DUTERTRE_ESTRADIOL_RESPONSE_24HR_UP [2], and DANG_MYC_TARGETS_UP [3], respectively. All signature files for this analysis were obtained from the GSEA website (www.broadinstitute.org/gsea/). Enrichment plots are used to visualize GSEA results. Enrichment scores (ES) and normalized p (NOM p) values were applied to the sorting pathways enriched after 1000 genome permutations above for analysis.

**GST pulldown assays**

GST and GST-WDR5 constructs were transformed into *E. coli*, DR5α strain. Protein expression and pulldown assays were performed using purified proteins and MCF-7 cell extracts expressing HA-PML1 according to our previous studies [4].

**Reverse transcription-quantitative PCR (RT-qPCR) analysis**

To quantify gene expression, we performed RT-qPCR using the Bio-Rad CFX-96 system and iQ SYBR Green Supermix kit (Bio-Rad, USA, #1708880) following the manufacturer's instructions. Primers for qPCR were designed using Primer Bank (http://pga.mgh.harvard.edu/primerbank/index.html) or NCBI-Primer Blast for ChIP. Each experiment was performed in triplicate or more, as indicated in Figure Legends, and the results were analyzed from three independent experiments.

**Chromatin immunoprecipitation followed by sequencing (ChIP-seq)**

ChIP were performed using the MCF-7 cell line, and MEFs were utilized as a spike-in control. For MCF-7 cells, shRNA-mediated knockdown of PML (shRNA-PML) or a control (shRNA-Ctrl) was accomplished. Subsequently, cellular cross-linking was achieved using 1% formaldehyde for 10 minutes at room temperature. Nuclei were isolated utilizing a hypotonic buffer (20 mM Tris, pH 7.4, 2 mM MgCl2, 5% glycerol) and subsequently lysed using nuclear lysis buffer (20 mM Tris, pH 7.4, 150 mM NaCl, 1% NP40, 0.5% DOC, 0.1% SDS, 1 mM EDTA). Chromatin was subjected to sonication, generating fragments within the range of 200-700 bp. Antibody pre-equilibration with beads (Dynabeads, Thermo Scientific), involving 2 μg for ChIP and 10 μg for ChIP-Seq, was performed overnight prior to a 6-hour immunoprecipitation of chromatin. Following rigorous washing, the bound chromatin was eluted using ChIP elution buffer (50 mM Tris, pH 8.0, 1 mM EDTA, 1% SDS, 50 mM NaHCO3), and subsequent protein digestion was conducted using 10 mg/ml proteinase K at 55°C for 1 hour. Chromosomal DNA was extracted using the QIAquick PCR Purification Kit (cat. 28104; Qiagen) and subjected to either quantitative PCR (qPCR) analysis or submitted for high-throughput sequencing after RNA digestion.

**ChIP-Seq analyses**

ChIP-seq analyses integrated both proprietary in-house datasets (GSE255018) and publicly available ENCODE portal data from researchers including Richard Myers (HAIB, 2012), Michael Snyder (Stanford, 2018), Vishwanath Iyer (UTA, 2012), and Bradley Bernstein (Broad, 2014) [https://www.encodeproject.org/]. The selected datasets encompassed ENCSR000BUZ (PML), ENCSR463GOT (ESR1), ENCSR000DMJ (MYC), and ENCSR985MIB (H3K4me3). Initial quality assessment of raw fastq files was conducted through Fastqc analysis. Subsequent data preprocessing involved Trim-galore (v0.6.5) for trimming and adapter removal, followed by genome alignment using Bowtie2 (v2.5.2). SPIKER (v1.0.0) was utilized to calculate scaling factors from the Spike-in control, and these factors were used to normalize ChIP profile using SPIKER and deepTools 2.0. For a more detailed exploration of chromatin-associated signals, deepTools generated informative heatmaps and metaplots. Peaks were characterized, and motif exploration was carried out using HOMER (v4.11), enabling comprehensive annotation

and analysis. Visualization of ChIP-seq outcomes was achieved through the Integrative Genomics Viewer (IGV) [5]. The MEME-suite Discriminative mode was employed to identify shared motifs between PML and ESR1.

**Cell invasion assays**

The invasion assay was performed using a Transwell chamber (8-μm pore size; Corning Inc., NY, USA) coated with collagen type I (50 μg /ml). $1 \times 10^5$ cells/well were seeded into the upper chamber of transwell inserts (24-well inserts) in serum-free medium. DMEM supplemented with 10% FBS was added to the bottom chamber. Cells were incubated for 48 h at 37 °C in a 5% CO2 incubator. After incubation, non-invasive cells were gently removed using a cotton swab. Cells that invaded the bottom chamber were fixed with 4% paraformaldehyde for 10 min, stained with 0.2% crystal violet, counted, and photographed under a light microscope.

**Colony formation assay**

For soft agar colony formation assay, 6-well plates were coated with a bottom layer of 1.2% SeaPlaque low melting temperature agarose (Lonza Rockland, ME USA) in DMEM supplemented with 20% FBS. Then, 2,000 cells were mixed in 0.6% agarose and the same medium and applied as the top agarose layer. The top agarose layer was overlaid with 600 μl medium. Plates were incubated at 37 °C in 5% CO2 for three weeks until colonies formed. At the end of the experiment, cell colonies were fixed with 4% paraformaldehyde and stained with 0.2% crystal violet (Sigma-Aldrich, #46364). The number of colonies was counted under a Nikon ECLIPSE Ts2R microscope, and pictures were taken. The assay was performed three times in triplicate.

**Cell proliferation and $IC_{50}$ assays**

MCF-7 cells infected with shRNA were seeded at a density of 1500 cells per well in 96-well plates. For the proliferation assay, cell numbers were assessed using the Cell Counting Kit-8 (CCK-8) (GLPBIO; Catalog No. GK10001) on days 0, 3, 5, and 7. In the IC50 assay, cells were seeded at the same initial density and cultured in media with or without fulvestrant or WDR5i (the drug concentrations varied in different experiments, as described in the figure legend), with medium changes every two days. Cell numbers were determined by CCK-8 on days 0

and 7. Optical density (OD) values at 450 nm were measured using a SpectraMax M2 plate reader. The growth rate was normalized, and data were plotted using GraphPad Prism 9.

**Immunoblot and co-immunoprecipitation**

Immunoblot protein analysis was performed according to standard protocols. For selected experiments, MCF-7 cells were incubated with $E_2$ and were harvested at the indicated time points before subsequent analyses. For co-IP, MCF-7 cells were lysed by Non-denaturing lysis buffer (20 mM Tris HCl pH 8, 137 mM NaCl, 10% glycerol, 1% Nonidet P-40 (NP-40), 2 mM EDTA), and the lysates were cleared by incubation with 50% protein A Sepharose bead slurry, after which 1ml of the cleared lysates were incubated with 50% antibody-conjugated protein A-Sepharose beads and 10 ul of 10% BSA overnight at 4ºC. The beads were washed three times with washing buffer (0.5% NP-40, 0.1% Triton X-100, 1 mM PMSF, and 1 mM Na3VO4 in PBS). Immunoprecipitates were subjected to western blots with indicated antibodies and immunoblotted using ECL detection kits (K-12045-D50; Laboratory Products Sales, NY, USA) and imaged by ChemiDoc MP imaging system (Bio-Rad).

***In-silico* analysis**

The data pertaining to PML protein interaction partners was obtained from the BioGRID database (http://www.thebiogrid.org), specifically, release version 4.4. The dataset included 725 interactions with PML in *Homo sapiens*. To cluster and annotate PML-interacting partners, we employed the Database for Annotation, Visualization, and Integrated Discovery (DAVID). Additionally, the enrichment plots were generated using the 'enrichplot' package (version 3.17) in R (version 4.3).

**Orthotopic breast cancer xenograft model**

NOD-SCID mice, aged 4–6 weeks, were bred under pathogen-free conditions at the Athymic Animal Core Facility of the Case Comprehensive Cancer Center. To assess the tumorigenic potential of PML1-expressing MCF-7 cells and control cells, 1 x $10^6$ cells were suspended in a mixture of PBS and Matrigel (100 µl, 1:1, BD Biosciences). The cell mixture was orthotopically injected into both sides of the fourth mammary fat pad of female NOD-SCID mice

(n = 10/group). Additionally, a subcutaneous inoculation of a 17β-estradiol pellet (Cat. No. SE-121 1.7 mg/pellet 60-day release; Innovative Research of America) was performed. Tumor volume (V) was measured using digital calipers with the formula: Width2 × Length/2. Once the tumor volumes reached over 125mm3, the xenografted mice were randomly divided into two groups: fulvestrant (5 mg/mice) and placebo (PBS) groups (n=10/group). Fulvestrant or vehicle was intraperitoneally injected into the mice twice per week. After eight weeks of inoculation or treatment, mice were euthanized by CO2 inhalation, and tumors were collected, weighed, and stored at -80°C for further analysis. All animal studies were conducted with the approval of the CWRU IACUC (2019-0040).

**TCGA data processing**

The Cancer Genome Altas (TCGA) Research Network datasets: http://gdac.broadinstitute.org/ were used to analyze the expression of specific genes in human tumors. In TCGA, 794 ER+ BC cases were analyzed. Survival curves were plotted using the Kaplan-Meier method and compared using the log-rank test using the Prism 9 software (GraphPad Software, CA, USA).

**Proximity Ligation Assay (PLA)**

Proximity Ligation Assay (PLA), employing the Duolink II Fluorescence kit from Olink Bioscience, was employed to investigate protein-protein interactions, following the manufacturer's stipulated protocols. Briefly, confluent adherent cells cultivated on 4-well chamber slides were fixed for 10 minutes using 4% paraformaldehyde. Subsequently, permeabilization was accomplished by treating the cells with 0.1% Triton X-100 in PBS for 10 minutes at room temperature, followed by blocking using 2% bovine serum albumin in PBS for 1 hour at the same temperature. To probe the interactions, we incubated cells overnight at 4°C with a pair of primary antibodies against PML and WDR5, ER, or a mouse IgG control. Post-primary antibody incubation, the slides underwent triple washes with 0.1% Triton X-100 in PBS, followed by a 1-hour incubation with two PLA probes (PLA probe MINUS stock and PLA probe PLUS stock; Duolink II) at room temperature. Another set of triple washes with 0.1% Triton X-100 in PBS was performed. The ligation step ensued, conducted for 30 minutes at 37°C, and succeeded by two washes using 0.1% PBS and Triton X-100. Subsequent amplification was facilitated by employing DNA polymerase

(Duolink II) for 100 minutes at 37°C. The slides were then meticulously washed, air-dried, and finally mounted with Dapi-Fluoromount-G (Southern Biotech). Subsequent imaging was conducted utilizing an OLYMPUS fluorescence microscope.

**Supplementary Tables and Figures**

**Table S1. Chemicals and antibodies used in this study.**

**Table S2. A list of primers and their sequences used in this study.**

**Table S3. ChIP-seq analyses of PML, ER, and Myc suggest the binding status of a list of stemness gene promoters.**

**Fig. S1. Expression profiles of *PML* isoforms in breast cancer subtypes. *A-B***, Expression levels of *PML* mRNA isoforms in normal (***A***), total breast cancer tissues (***B***), ER+ (***C***), HER2+ (***D***), and basal breast cancer (***E***). ***F***, Relative abundance of *PML* isoforms (UCSC genome browser, hg38) breast cancer subtypes.

**Fig. S2. *A*,** The prognosis values of *PML* isoforms expression. ***B***, Higher PML protein levels correlate with poor prognosis, as illustrated in the plot generated by KMplot.com (http://kmplot.com/analysis/index.php?p=background). The data for ER+ patients were retrieved from the Tang_2018 database [6].

**Fig. S3. The *PML* gene is amplified in 14% of ER+ breast tumors.**

**Fig. S4. A,** PML4 does not rescue the proliferation of *PML* knockdown cells. MCF-7 cells were transiently transfected with *PML* shRNA followed by transfection with vector, PML1, or PML4 expression plasmids,

and cell numbers were determined five days after transient transfection of PML1 or PML4. ***B,*** PML2 inhibits the proliferation (left) and stemness (right) of MCF-7 cells.

**Fig. S5. Images of tumorspheres in PML1 overexpression and *PML* knockdown MCF-7 and ZR-75-1 cells.**

**Fig. S6.** ***A,*** Heatmaps of ER- and PML-binding sites on ER-bound promoters. ***B,*** The top consensus sequence motif of PML1- and ER-shared promoters. ***C,*** PML target genes are enriched in Myc target genes.

**Fig. S7**, ChIP-seq tracks show the binding sites of PML, ER, and Myc on *JAG1*, *KLF4*, *MYC*, *SNAI1*, and *YAP1* genes. The red line marks TSS.

**Fig. S8. Proximity ligation assays detection of interactions between PML and ER and PML and WDR5.**

**Fig. S9. Images of *WDR5* knockdown tumorspheres.**

**Reference**


1 Hu Y, Smyth GK. ELDA: extreme limiting dilution analysis for comparing depleted and enriched populations in stem cell and other assays. *J Immunol Methods* 2009; 347: 70-78.

2 Dutertre M, Gratadou L, Dardenne E, Germann S, Samaan S, Lidereau R *et al*. Estrogen regulation and physiopathologic significance of alternative promoters in breast cancer. *Cancer Res* 2010; 70: 3760-3770.

3 Zeller KI, Jegga AG, Aronow BJ, O'Donnell KA, Dang CV. An integrated database of genes responsive to the Myc oncogenic transcription factor: identification of direct genomic targets. *Genome Biol* 2003; 4: R69.

4 Hsu KS, Kao HY. beta-Transducin repeat-containing protein 1 (beta-TrCP1)-mediated silencing mediator of retinoic acid and thyroid hormone receptor (SMRT) protein

degradation promotes tumor necrosis factor alpha (TNFalpha)-induced inflammatory gene expression. *J Biol Chem* 2013; 288: 25375-25386.

5 Robinson JT, Thorvaldsdottir H, Winckler W, Guttman M, Lander ES, Getz G *et al*. Integrative genomics viewer. *Nat Biotechnol* 2011; 29: 24-26.

6 Tang W, Zhou M, Dorsey TH, Prieto DA, Wang XW, Ruppin E *et al*. Integrated proteotranscriptomics of breast cancer reveals globally increased protein-mRNA concordance associated with subtypes and survival. *Genome Med* 2018; 10: 94.
