# Supplementary figures and images for: The PML1-WDR5 axis regulates H3K4me3 marks and promotes stemness of estrogen receptor-positive breast cancer
Source: Cell Death Differ. 2024 Apr 16;31(6):768–78. doi: 10.1038/s41418-024-01294-6 (PMC11164886; doi:10.1038/s41418-024-01294-6)

Fig. S1

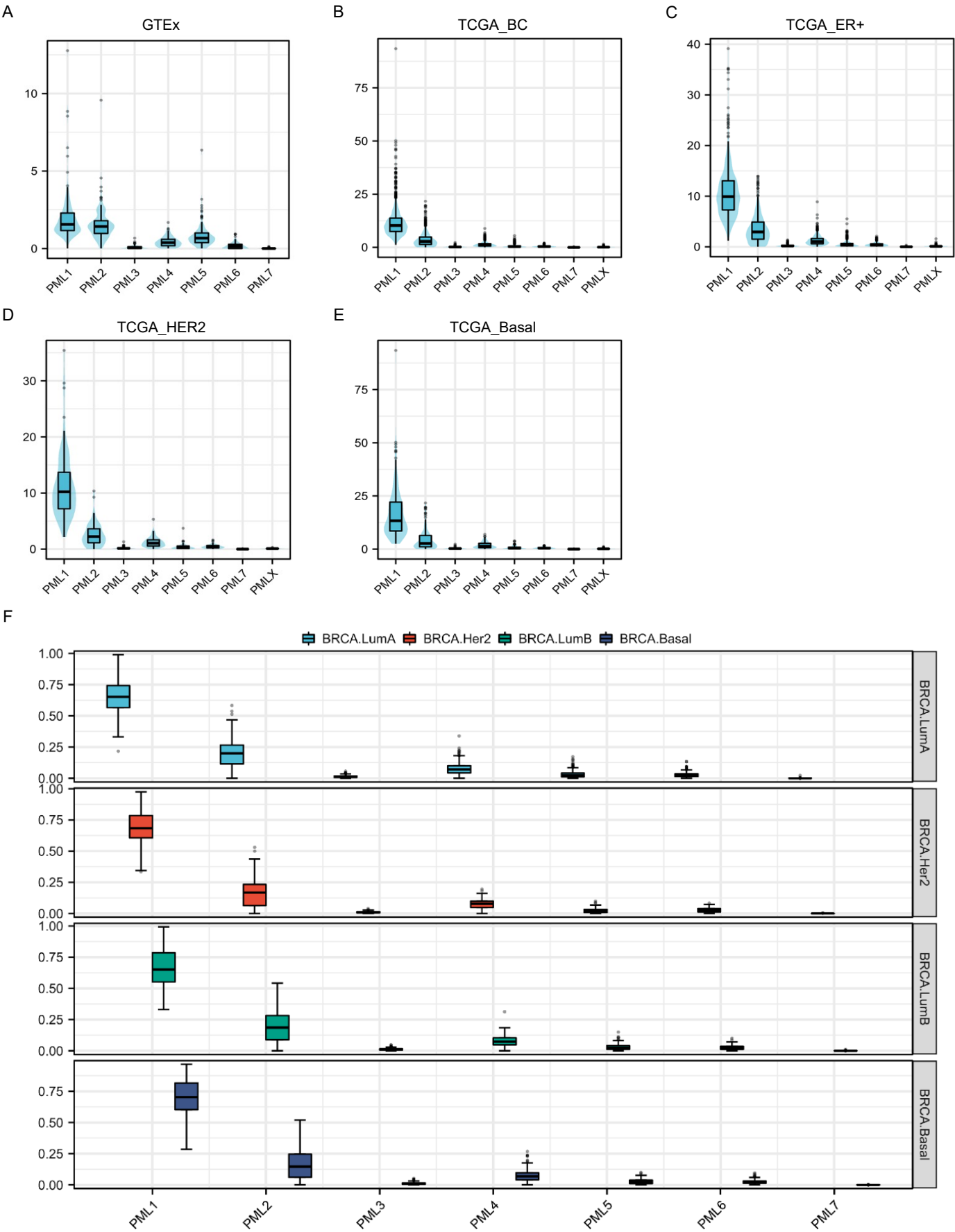

Fig. S2

A

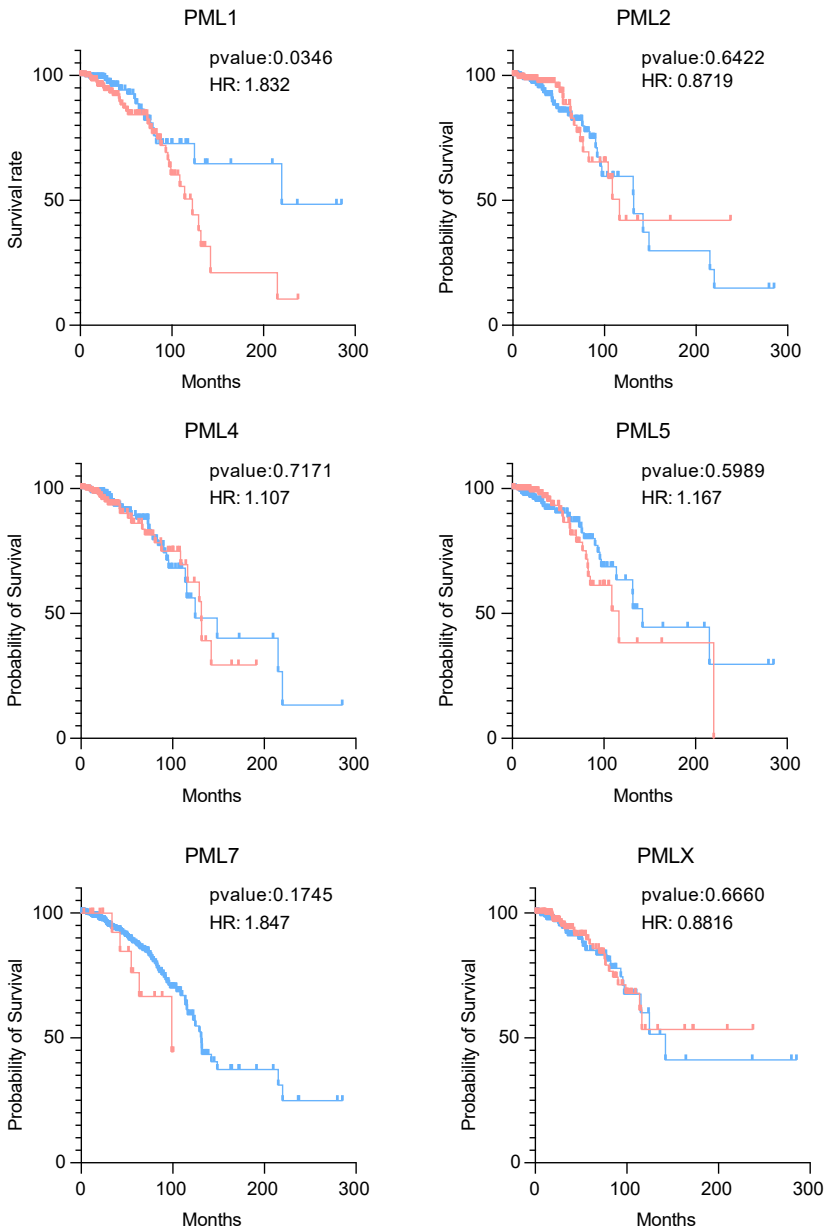

B

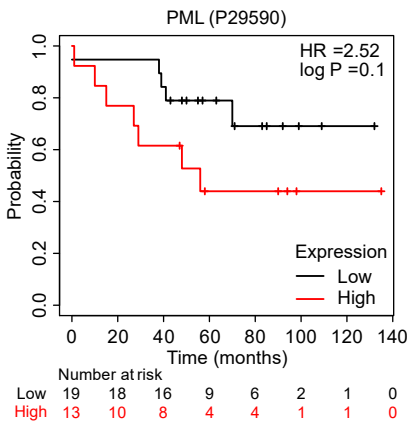

Fig. S3

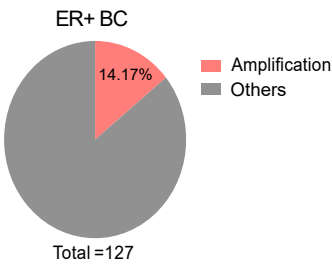

Fig. S4

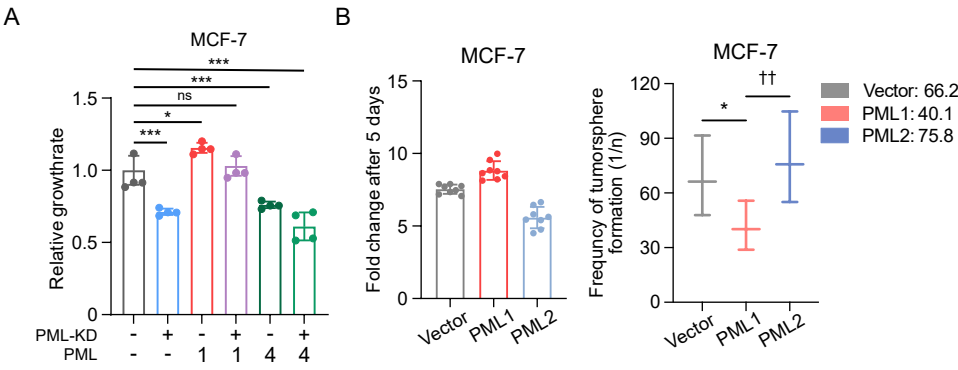

Fig. S5

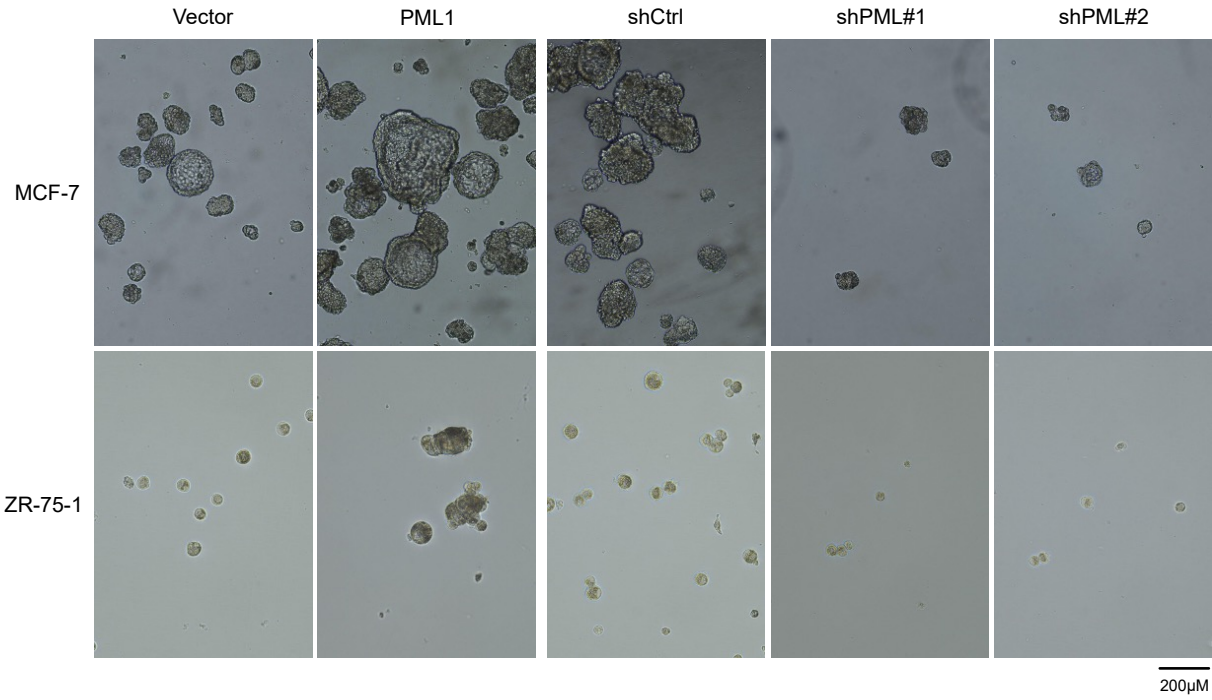

Fig. S6

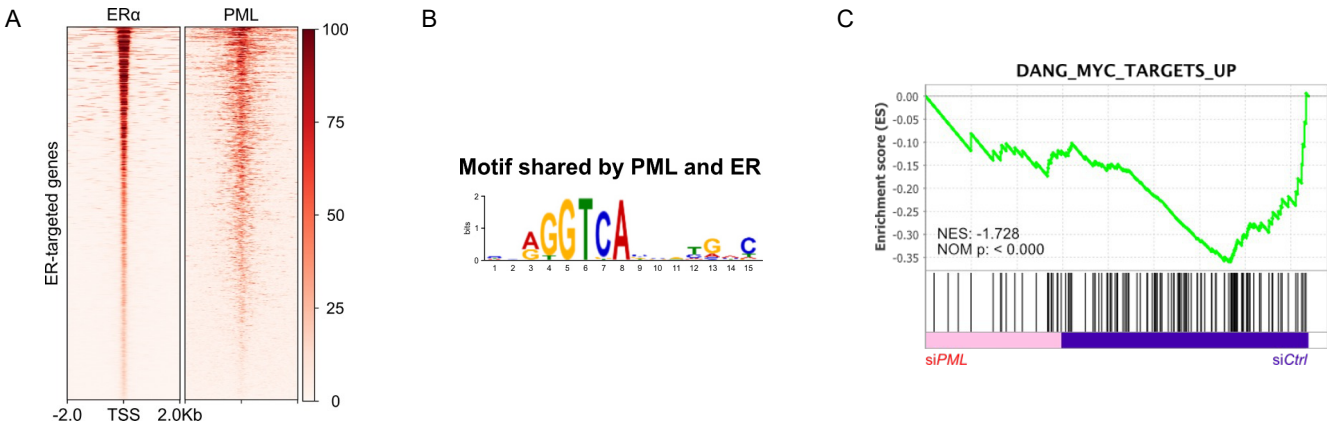

Fig. S7

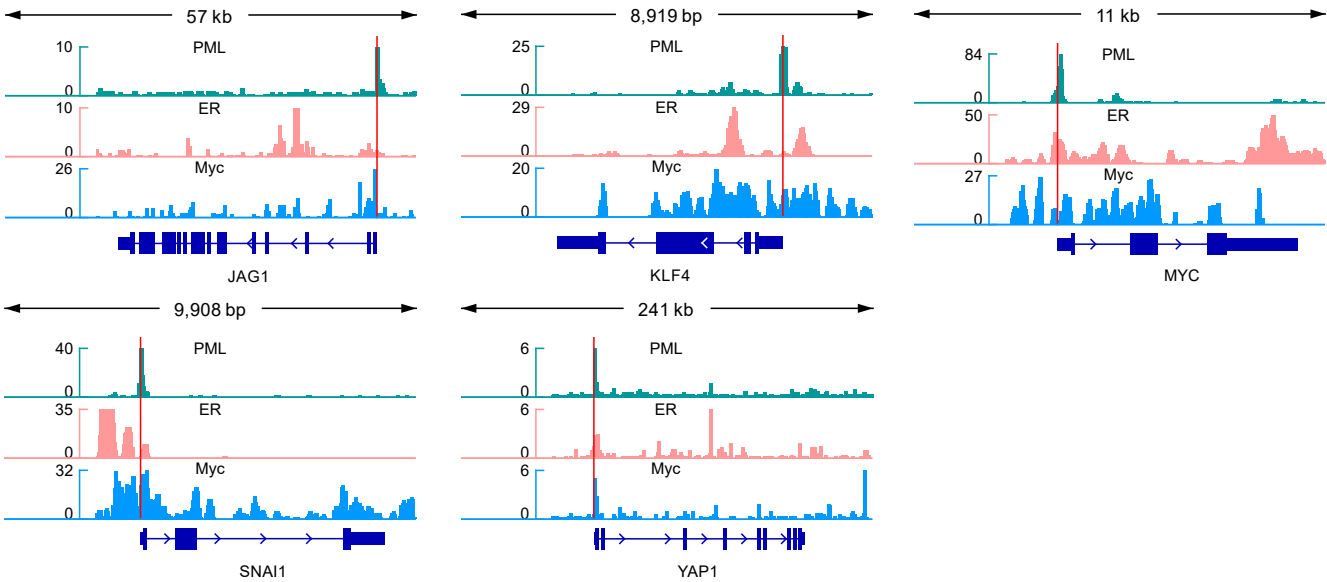

Fig. S8

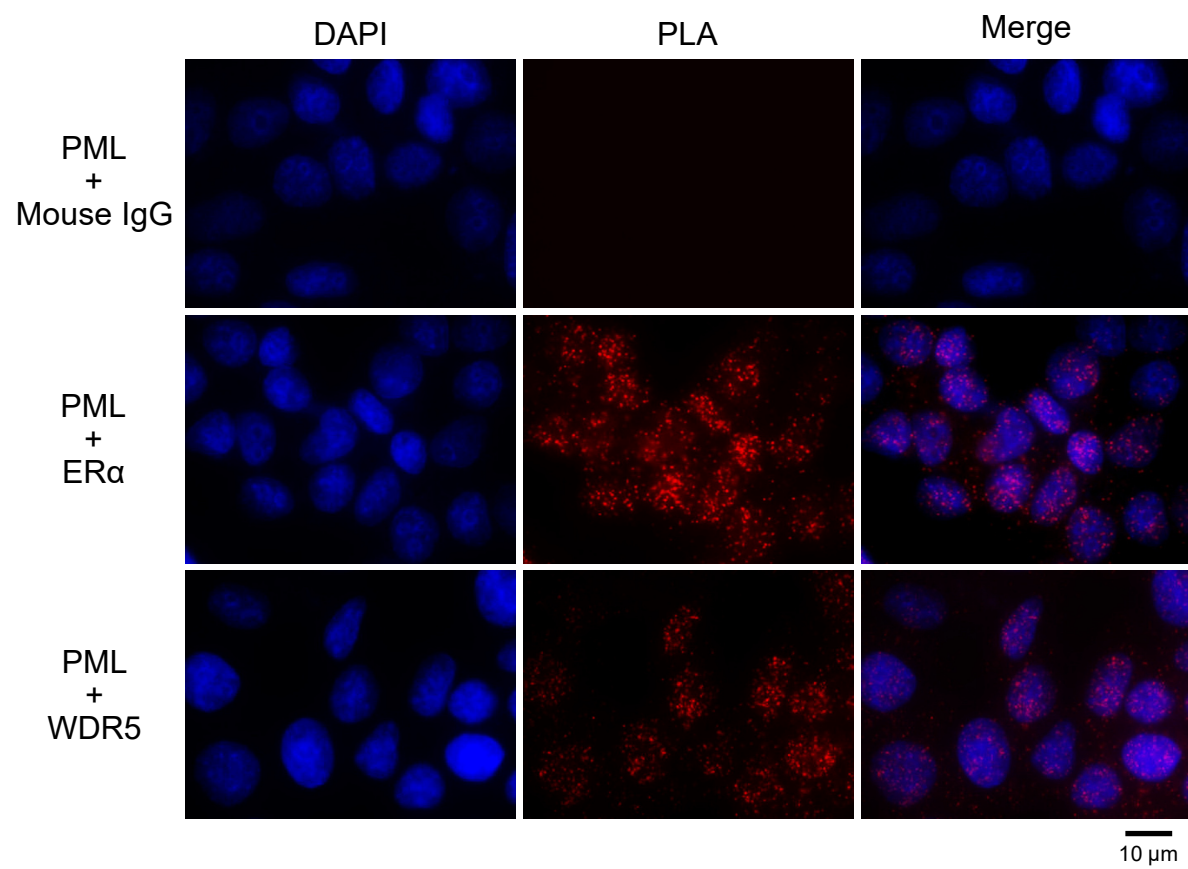

Fig. S9

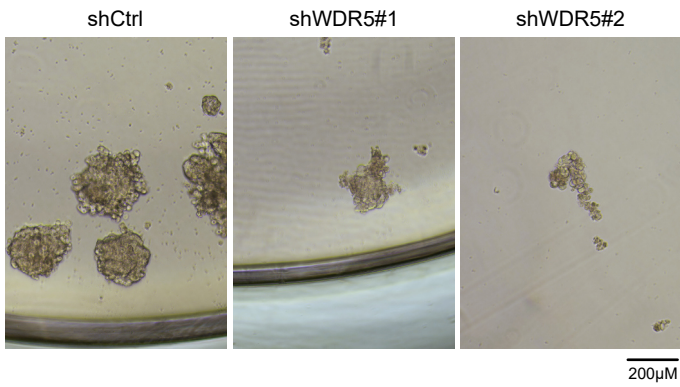

Supplement: Supplementary file 2 — Supplementary Figures [file 41418_2024_1294_MOESM2_ESM.pdf]
