# Supplementary material for: The PML1-WDR5 axis regulates H3K4me3 marks and promotes stemness of estrogen receptor-positive breast cancer
Source: Cell Death Differ. 2024 Apr 16;31(6):768–78. doi: 10.1038/s41418-024-01294-6 (PMC11164886; doi:10.1038/s41418-024-01294-6)

Fig. S10

Fig1F

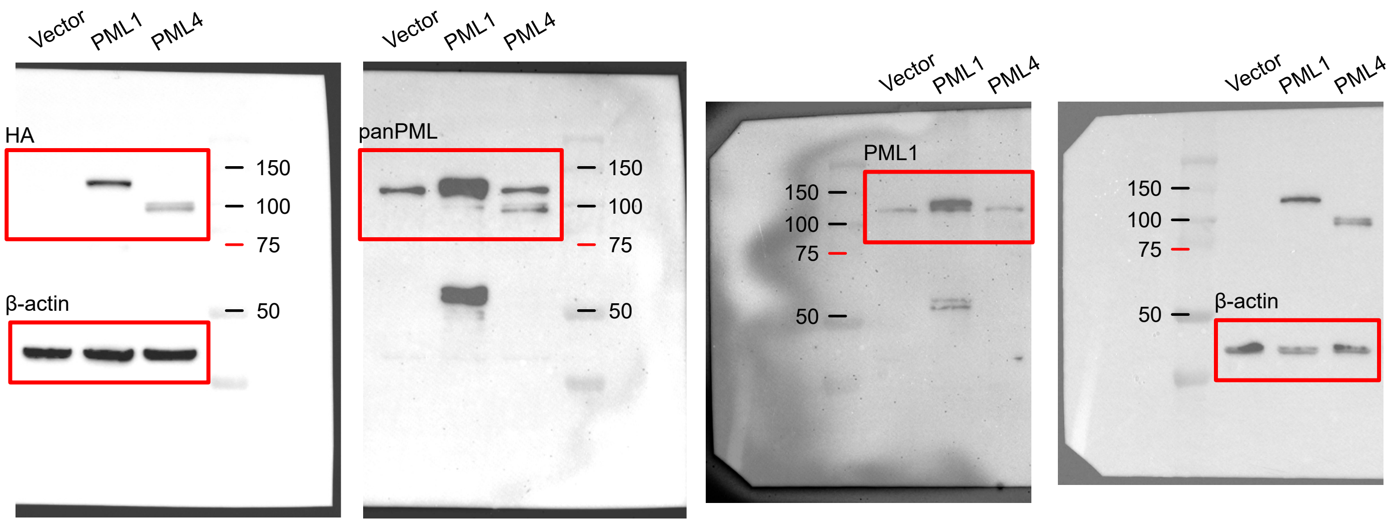

Fig1G

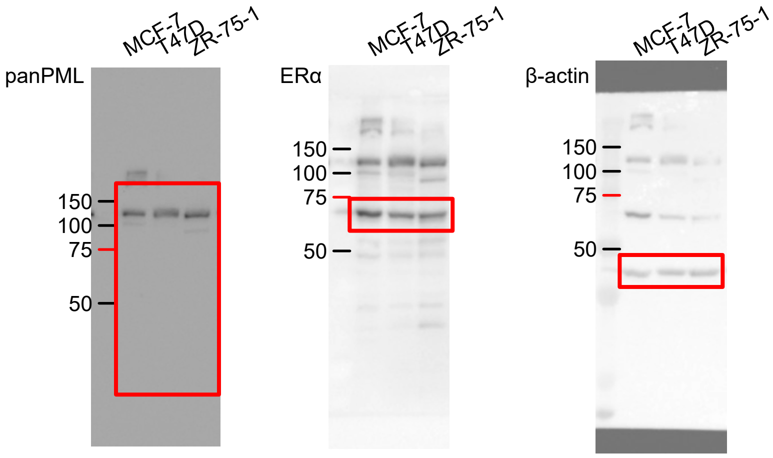

Fig. S10

Fig. 2A

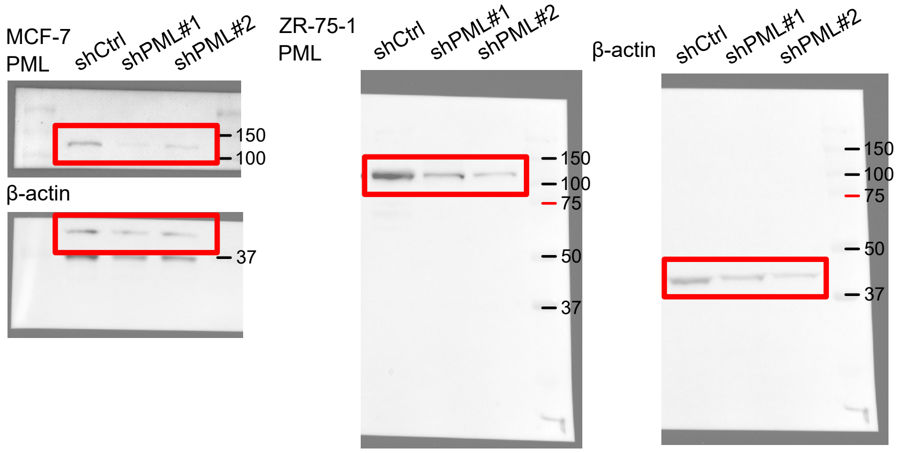

Fig. 2B

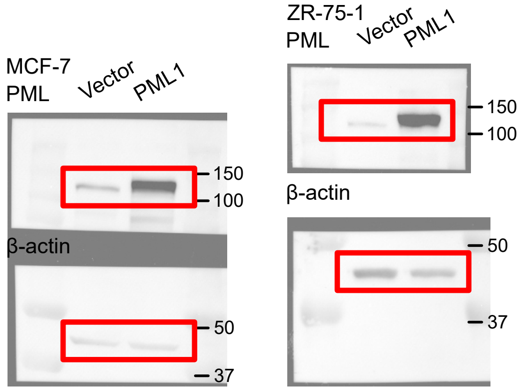

Fig. 2C

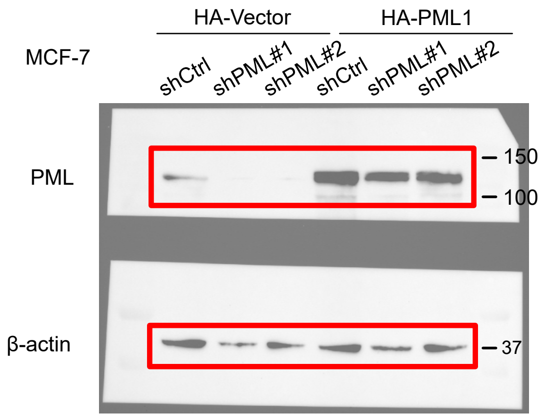

Fig. S10

Fig. 4A

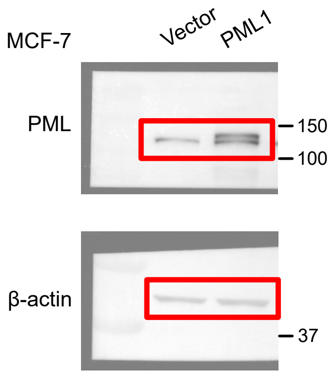

Fig. 4C

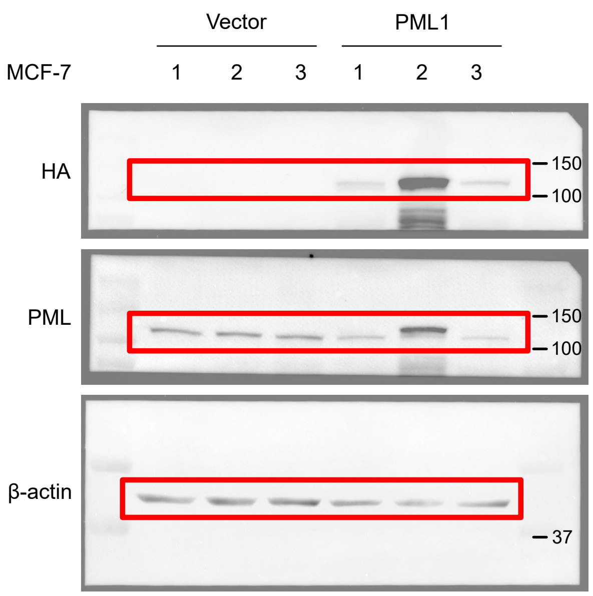

Fig. S10

Fig. 5I

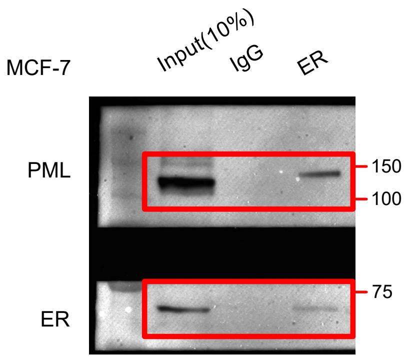

Fig. 5J

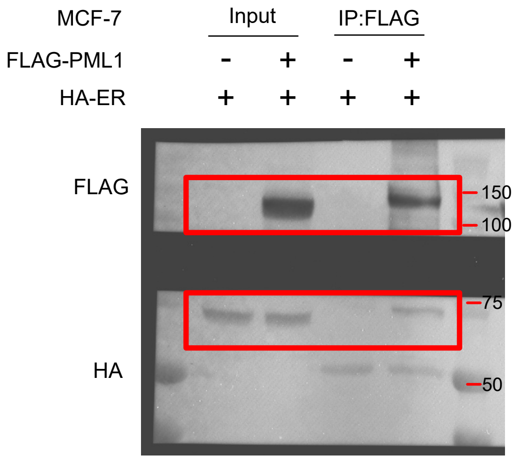

Fig. 5K

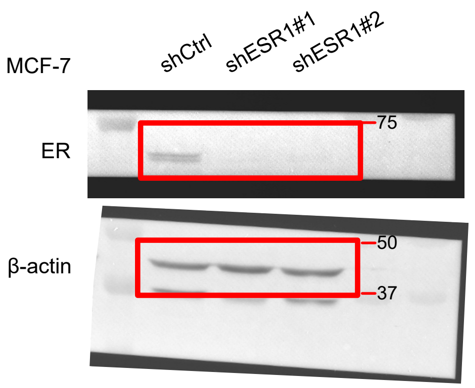

Fig. S10

Fig. 6C

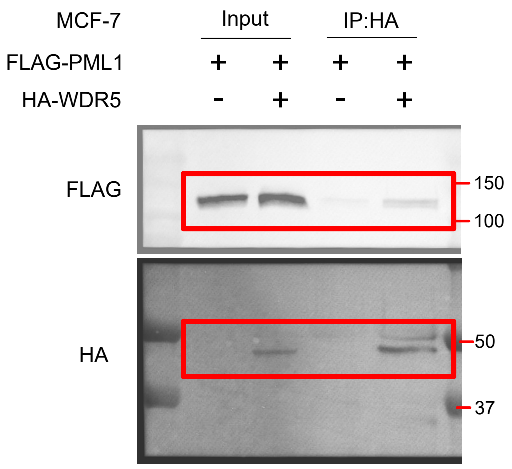

Fig. 6D

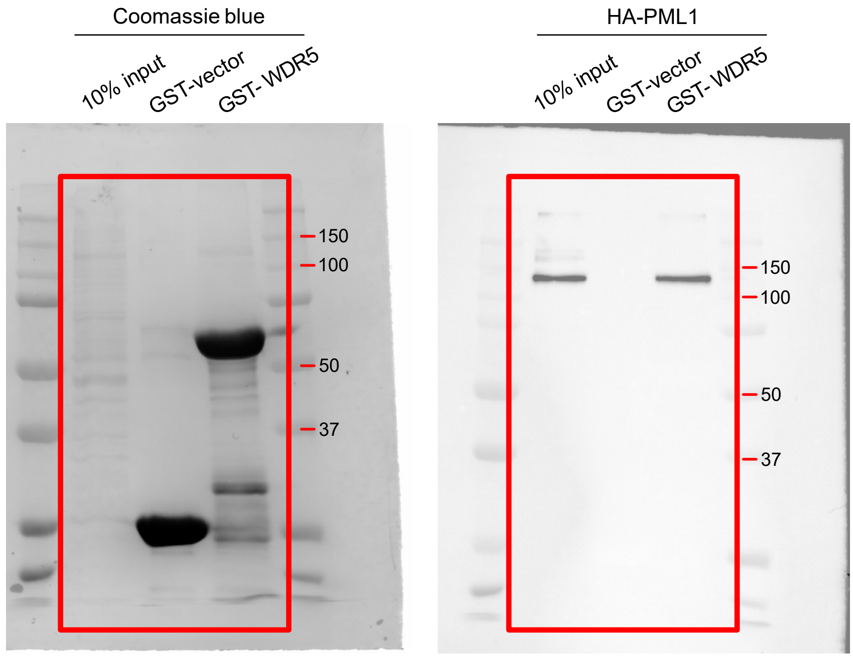

Fig. S10

Fig. 7A

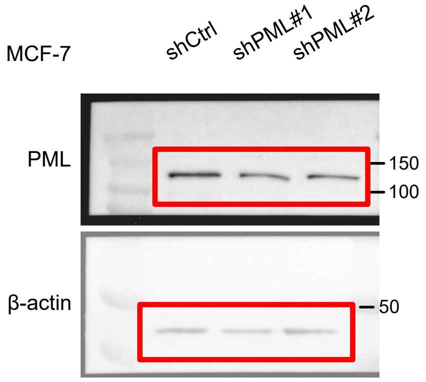

Fig. 7C

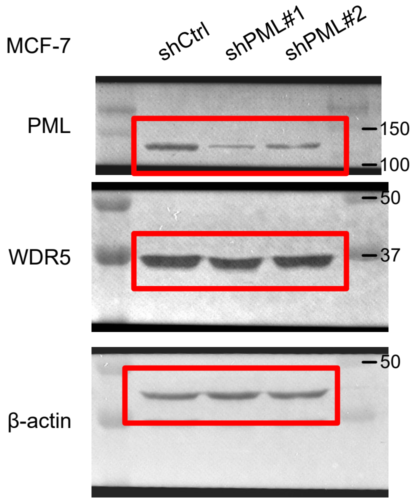

Fig. 7H

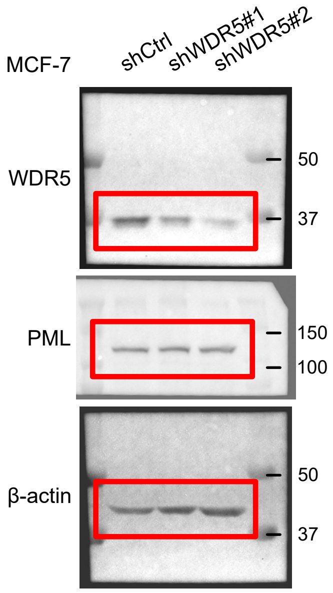

Supplement: Supplementary file 4 — Original Western Blot [file 41418_2024_1294_MOESM4_ESM.pdf]
